# Surface Engineering of AgNPs-Decorated Polyetheretherketone

**DOI:** 10.3390/ijms24021432

**Published:** 2023-01-11

**Authors:** Jakub Siegel, Barbora Vyhnálková, Tatiana Savenkova, Jana Pryjmaková, Petr Slepička, Miroslav Šlouf, Tomáš Hubáček

**Affiliations:** 1 Department of Solid State Engineering, University of Chemistry and Technology Prague, 166 28 Prague, Czech Republic; 2 Institute of Macromolecular Chemistry, Academy of Sciences of the Czech Republic, Heyrovského nám. 2, 162 06 Prague, Czech Republic; 3 Biology Centre of the Czech Academy of Sciences, SoWa National Research Infrastructure, Na Sádkách 7, 370 05 České Budějovice, Czech Republic

**Keywords:** laser treatment, periodic structures, silver nanoparticles, bactericidal effect, surface morphology

## Abstract

Metal nanostructure-treated polymers are widely recognized as the key material responsible for a specific antibacterial response in medical-based applications. However, the finding of an optimal bactericidal effect in combination with an acceptable level of cytotoxicity, which is typical for metal nanostructures, prevents their expansion from being more significant so far. This study explores the possibility of firmly anchoring silver nanoparticles (AgNPs) into polyetherether ketone (PEEK) with a tailored surface morphology that exhibits laser-induced periodic surface structures (LIPSS). We demonstrated that laser-induced forward transfer technology is a suitable tool, which, under specific conditions, enables uniform decoration of the PEEK surface with AgNPs, regardless of whether the surface is planar or LIPSS structured. The antibacterial test proved that AgNPs-decorated LIPSS represents a more effective bactericidal protection than their planar counterparts, even if they contain a lower concentration of immobilized particles. Nanostructured PEEK with embedded AgNPs may open up new possibilities in the production of templates for replication processes in the construction of functional bactericidal biopolymers or may be directly used in tissue engineering applications.

## 1. Introduction

Significant progress has been made in the field of optomechanical manipulation with metal nanoparticles (NPs) in the past decade [1,2,3,4,5]. In addition to optical tweezers [1,6], which allows precise manipulation with a single molecule or tiny parts of condense matter—especially diverse nanoparticles—promising approaches nowadays are based on the collective movement of a large amount of such material [4,5]. These advanced procedures known as laser-induced forward transfer (LIFT) [7], if performed in eligible environments, allow the construction of composites that combine materials as diverse as polymers and metals on a robust dimensional scale. The unquestionable advantage of these materials is the very essence of the anchoring of metal nanoparticles in a polymer matrix, which has a purely physical nature. The absence of a chemical interlayer, which is necessary in conventional surface grafting approaches, opens up unprecedented application strategies in areas such as targeted drug delivery [8,9], separation and membrane processes [10,11] or biomedical sciences and engineering [4].

During directed movement of metal NPs, which is caused by the forward scattering force arising from the isotropic scattering of incoming electromagnetic radiation on spherical particles with sufficiently small diameter (typically less than 50–60 nm), the light absorption occurs at wavelengths close to the resonant conditions of metallic particles, leading to their simultaneous heating [4]. Once the glass transition temperature of the semicrystalline polymer is overcome, nanoparticles are pushed into the polymer surface, creating a composite with considerably high concentration of particles in a close vicinity of the surface.

LIFT technology has been successfully applied in the preparation of black metal absorbers [7] or advanced antimicrobial coatings [12]. At present, it is also quite challenging to control the specific microstructure of the prepared composites, which may crucially affect the properties of the incorporated metallic nanoparticles. For example, optical properties are critically dependent on the dielectric nature of the surrounding environment, which is sensitive to surface morphology [13]. In biomedical applications, the specific surface structure may promote cell adhesion and proliferation [14] or it may cause preferential orientation of cells on the culture substrate [15]. The microstructure also determines the specific surface area, which has a fundamental impact on the bactericidal properties of LIFT-processed polymeric surfaces [16] or the transport and retention properties in membrane and separation processes [17]. With respect to this, several studies showed that excimer laser light may effectively develop LIPSS (laser induced periodic surface structure) on a wide range of polymeric materials [18,19] once the polymer absorbs the specific wavelength of the irradiation beam.

Generally, laser-treated polymer materials offer exceptional surface characteristics that can be exploited for the production of nanocomposites, the main components of which are metal nanoparticles. In this way, a number of unique properties can be imparted to the polymer matrix, especially when silver NPs are applied. In biomedical applications, bactericidal activity is a highly desirable characteristic and silver in its various structural forms is one of the longest and most widely used antibacterial agents due to its efficacy, price, and industrial availability [20,21], while various forms of silver have been applied. Except for colloid solution, silver has a pronounced antibacterial effect as coating [22,23,24] or in the form of silver-based nanocomposites [25]. Today, many research papers refer to silver–polymer composites with bactericidal effect [26,27,28]; however, direct combination of silver and polyetheretherketone (PEEK) is still rare. As PEEK is an extended material in dentistry and bone tissue regeneration [29,30,31], challenging methods enabling long-term stability with strong binding of nanoparticles to polymer are desirable in construction of prospective materials. The findings presented in the works of Sanpo et al. [32] and Liu et al. [33], which introduce the implementation of AgNP near the surface of PEEK, seem to be the first attempts to preserve long-term antibacterial persistence with increased antibacterial effectivity for these types of composites.

Besides AgNPs/PEEK composites, other PEEK-based materials that are very promising from a bactericidal and biocompatibility point of view are being intensively researched these days. In addition, in the case of these materials, the main idea is the synergy between the built-in component acting as an active bactericidal agent and the specific surface microstructure, which can enhance the desired effects of these composites [34,35,36].

In this work, we focus on the engineering of the surface microstructure of PEEK prior to its decoration with silver nanoparticles (AgNPs). We took over parameters such as laser fluence during the LIPSS formation process to obtain the polymer surface evenly covered with coherent ripple structures and immobilization laser fluence to prevent destruction of those LIPSS during the polymer decoration process from our previous work on optimisation of those parameters on PET [5,18]. Promising PEEK biopolymer with precisely controlled surface morphology embedded with silver nanoparticles may open up new possibilities in the production of templates for further replication processes in the construction of functional bactericidal biopolymers or may be applied directly in tissue engineering due to the natural biocompatibility of PEEK.

## 2. Results and Discussion

**Figure 1:**
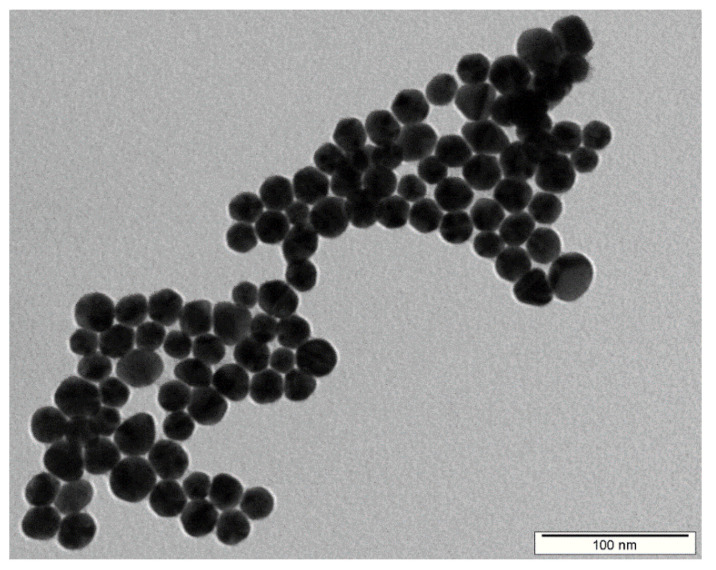
TEM image of as-synthesized AgNPs entering the immobilization process.

The immobilization of silver nanoparticles was performed from a solution of AgNPs synthesized by the electrochemical method. Figure 1 shows the TEM image of as-synthesized AgNPs entering the immobilization process. Their regular round shape is clearly identifiable with a low variable of sizes. The average particle size was determined to be 23.4 ± 4.2 nm.

**Figure 2:**
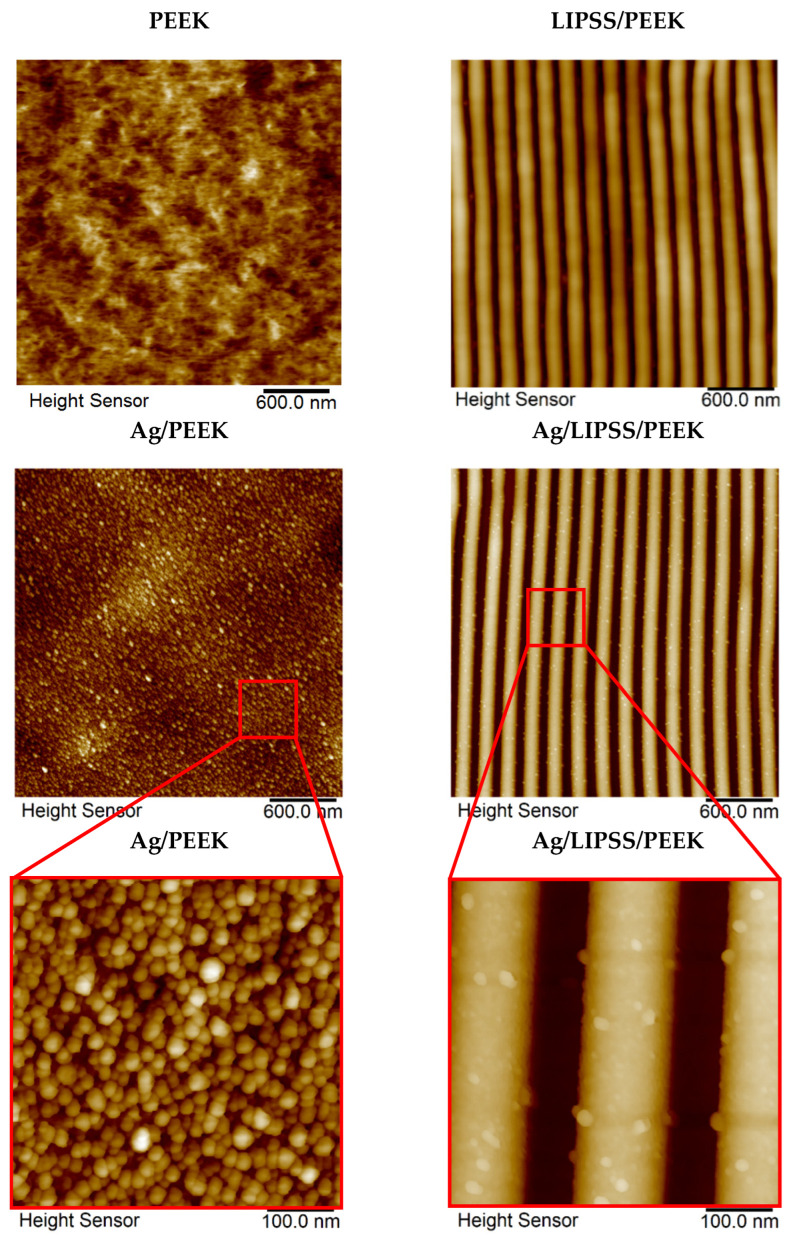
The 2D AFM images of as-received PEEK, PEEK with LIPSS structure (LIPSS/PEEK), AgNP-decorated PEEK (Ag/PEEK) and AgNP-decorated PEEK with LIPSS structure (Ag/LIPSS/PEEK) together with details of AgNP-decorated structures.

In terms of the final surface morphology of the prepared structures, we pursued two sub-goals in this work. First, we applied a laser immobilization fluence that causes the simple incorporation of AgNPs by LIFT technology into the surface without changing the naturally planar morphology of the polymer. Second, we used the same laser fluence during LIFT processing to the laser-pretreated PEKK with well-developed LIPSS structure to achieve a simple decoration of such structures with AgNPs without their damaging. Subsequently, we studied synergy of regular LIPSS with immobilized AgNPs. The surface morphology of laser-processed PEEK was studied by AFM. Figure 1 shows 2D AFM images of the pristine PEEK together with the laser-processed PEEK with the well-developed LIPSS structure before and after immobilization with AgNPs. It is obvious from Figure 2 that the as-received PEEK foil was pretty planar with considerably low surface roughness *R*_a_, while the laser-pretreated PEEK exhibited a regular surface grating known as LIPSS. It is obvious that originally planar PEEK was transformed into periodic structures, evenly spread over the polymer. Once the LIPSS is developed, the surface roughness increases dramatically. LIPSS formation is a quite complex process originating from the interaction of the incoming laser beam with the surface acoustic wave (SAW) as a consequence of light refraction in the vicinity of the polymer surface [37].

The most striking observation that emerges from Figure 2 is the successful decoration of PEEK with AgNPs. It is evident that the planar nature of the as-received PEEK (Ag/PEEK) and the regular pattern nanostructure of Ag/LIPSS/PEEK was preserved even after the immobilization process, and both structures were evenly decorated with AgNPs. After immobilization, the surface roughness increased slightly (Table 1). The inset shows details of the selected area of both structures, directly visualizing the immobilized AgNPs. As expected, when regular surface patterns (LIPSS) were formed, the surface roughness increased dramatically (see Table 1). Regardless of the nature of the underlying substrate (planar or LIPSS), the immobilization process causes a slight increase in surface roughness due to the incorporation of metal nanoparticles in the close vicinity of the polymer surface. Other surface characteristics, such as width *Λ* and height *h* related to the LIPSS nanostructure, are also presented in Table 1. It is obvious that the choice of immobilization fluence applied to LIPSS structures corresponding to that preserving planar morphology in the case of as-received PEEK was also useful in the case of LIPSS/PEEK samples, since the LIPSS structure was completely preserved after the immobilization process. This conclusion is supported by both the AFM images themselves and the surface characteristics of related samples (height and width), which remain practically unchanged after the immobilization process.

**Figure 3:**
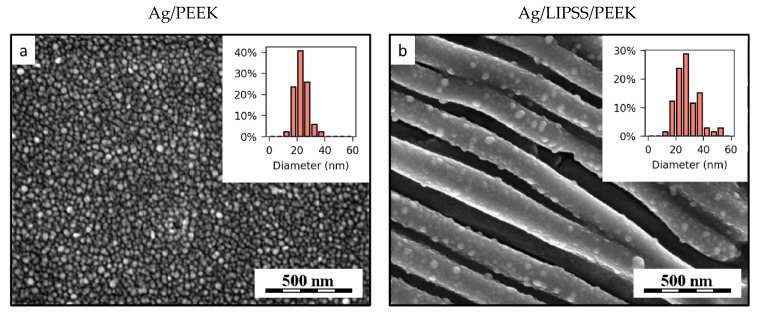
FEG-SEM micrographs of PEEK after immobilization with AgNPs (**a**) on as-received planar polymer (Ag/PEEK) and (**b**) on laser pre-treated polymer with LIPSS structure (Ag/LIPSS/PEEK). The insets in the upper right corner of both figures are histograms showing particle size distribution.

In addition to AFM analysis, we performed FEG-SEM observation of AgNP-decorated samples to better illustrate the distribution of nanoparticles on the polymer surface, which is possible due to considerable material contrast between polymer and AgNPs. FEG-SEM microphotographs are shown in Figure 3, together with histograms showing size distribution of immobilized particles. Similar to results of AFM analysis, one can clearly distinguish AgNPs on both the original flat PEEK surface and PEEK with LIPSS. It is evident that once the immobilization process is performed on PEEK with LIPSS, the concentration of metal particles is lower compared to that of the planar polymer. This could probably be due to the mechanism of LIPSS formation, which consists of redistribution and recrystallization of polymer matter under irradiation with polarized laser light [37]. However, the FEG-SEM images support the results of the AFM analysis, proving that the immobilization process itself does not break the LIPSS structure, which is evenly decorated with AgNPs. Nevertheless, we were unable to detect the area with strictly regular surface grating as in the case of AFM analysis (see Figure 2). The average particle diameters are 23.4 nm and 28.4 nm for Figures (a) and (b), respectively. The slightly higher apparent average size of the particles in Figure (b) could be attributed to the fact that the AgNPs were partially submerged in the polymer matrix, which made the smallest particles almost invisible.

**Figure 4:**
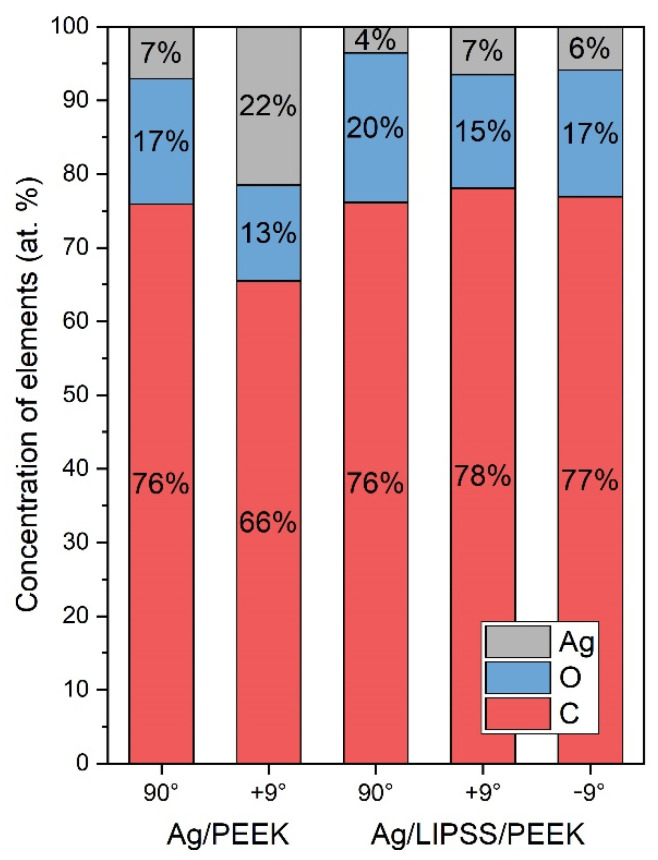
Concentration (at.%) of Ag (3d), O (1s), C (1s) on the surface of PEEK (Ag/PEEK) and PEEK with LIPSS structure (Ag/LIPSS/PEEK) after immobilization with AgNPs, measured by XPS. The analysis was carried out in a perpendicular (90°) and a tilted angle geometry (+9°, −9°).

As can be seen from both AFM and FEG-SEM images, the immobilization of AgNPs on the polymer surface with LIPSS most probably leads to a lower concentration of embedded nanoparticles in the polymer. Therefore, we performed XPS analysis to quantify the Ag content in the polymer after the immobilization process. The concentrations of silver (Ag), oxygen (O), and carbon (C) are summarized in Figure 4. Atomic concentrations of O and C in as-received PEEK derived from polymer stoichiometry are 15.3% and 84.7%, respectively. We performed the XPS measurement perpendicularly to the sample surface (90°), which provides analytical information from deeper areas of the AgNPs/PEEK composite (typically reaching ones of nanometers), and in the tilted geometry (9°), providing a composition of very surface area, typically from less than 10 atomic layers. In the case of AgNP-decorated LIPSS, we recorded the XPS signal from both sides (+9° and −9°) perpendicular to the LIPSS in order to confirm or exclude preferential immobilization of AgNPs on the surface with regular patterns.

As expected, the data in Figure 4 show that the concentration of silver in the planar polymer immobilized with AgNP (Ag/PEEK) almost doubles compared to the samples with LIPSS. While planar PEEK contained 7 and 22 at.% Ag under perpendicular or tilted angle geometry, laser pretreated PEEK with LIPSS structure exhibited 4 and 6 at.% Ag, respectively. Moreover, it is evident that under the present experimental setup, no preference for AgNPs immobilization occurs once the LIPSS structure is present. Slight increase in O concentration in the case of LIPSS samples may be attributed to the reorientation of the polar groups in the surface layers during LIPSS formation and thus the oxygen content may be higher in comparison with as-received PEEK. Similar phenomenon has been observed also in the case of PET [18].

Because of the potential use of our newly developed structures in the replication process of biomimetic materials or as carriers with a specific antibacterial response in tissue engineering applications, it was essential to characterize the anchorage strength between AgNPs and the polymer carrier. Anchorage strength was evaluated by leachate tests for the two model cases, (i) in static contact with the water environment and (ii) under dynamic conditions in the same environment.

**Figure 5:**
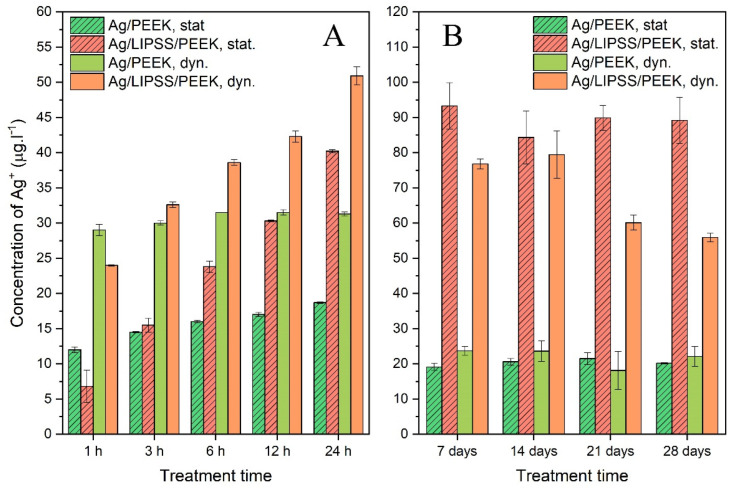
Concentration of Ag ions in leachates after treatment of Ag-immobilized samples in water determined by the ICP-MS technique for short-term (**A**) and long-term (**B**) experiments.

Figure 5 summarizes the Ag concentrations detected in leachates using the ICP-MS technique in short-term (A) and long-term (B) experiments. As expected for short-term experiments, it generally rules that, in dynamic exposition, AgNPs are released more willingly compared to the static one. Whereas in the case of planar samples (Ag/PEEK) the saturation of leachate with Ag ions occurred practically immediately under dynamic conditions (within one day), leachates from the samples with nanostructured morphology (Ag/LIPSS/PEEK) saturated in as long as one week. This is most likely caused by the presence of a regular pattern nanostructure, which prevents fluid flow in the immediate vicinity of the surface; this is particularly true also for planar samples under static conditions in short-term experiment. Surprisingly, in the case of Ag/PEEK, the initial saturation of leachates with Ag ions at a level of 30 μg·l^−1^ gradually decreases in long-term treatment to 20 μg·l^−1^. The same trend was observed in the case of Ag/LIPSS/PEEK under dynamic conditions. We do not yet have a satisfactory explanation for this phenomenon, but it could probably be related to some kind of passivation of silver structures. Generally, the presence of LIPSS increases the specific surface area that affects the concentration of Ag in leachates that more than quadruples compared to planar samples (approximately 85 and 20 μg·l^−1^ for planar and LIPSS structure, respectively), regardless of specific treatment conditions (static or dynamic). It may be concluded that Ag-immobilized PEEK foil serves as a long-term source of Ag ions in a liquid environment, when the specific surface morphology directly affects the saturation capacity.

Furthermore, we determined the total amount of biologically available silver (TABAAg), defined as a concentration of Ag acquired by analysis of a nitric acid-immersed sample with immobilized AgNPs. The TABAAg content was determined to be 956 and 894 μg·L^−1^ in the case of Ag/PEEK and Ag/LIPSS/PEEK, respectively. The slight discrepancy of these two values may be due to the shadow effect of LIPPS, which probably prevents all AgNPs from being dissolved.

**Figure 6:**
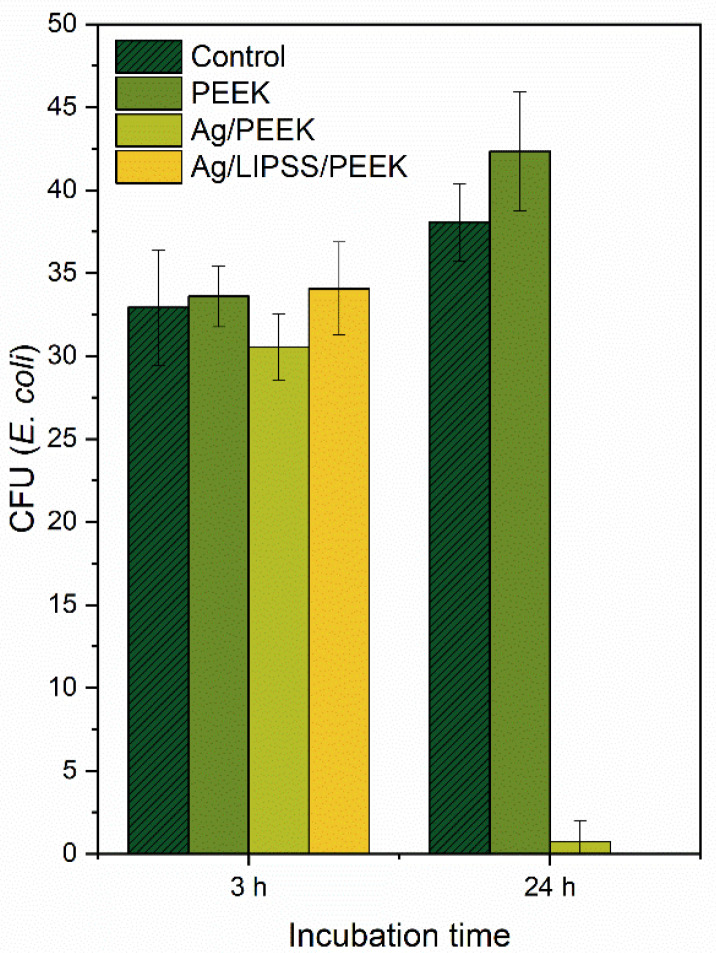
Viability of *E. coli* for different incubation times with Ag-treated PEEK expressed by the number of CFU, determined by the drop plate test.

The antibacterial activity of Ag–polymer nanocomposites was determined by drop plate tests conducted on PEEK, Ag/PEEK, and Ag/LIPSS/PEEK. *E. coli* was chosen as the common pathogen of Gram-negative bacteria that cause infections and biofilm colonization [38]. The results are shown in Figure 6.

As expected, no evidence of bacteria inhibition was detected in the case of PEEK after 3 h of incubation. Furthermore, after 24 h of incubation, the count of bacteria in as-received PEEK increased slightly, which can be explained by the gradual adaptation of the bacteria. Moreover, both AgNP-immobilized samples (planar—Ag/PEEK and nanostructured—Ag/LIPSS/PEEK) did not show antibacterial effect after 3 h of incubation. The most striking result that emerges from Figure 6 is that the CFU of *E. coli* decreased considerably for Ag/PEEK and Ag/LIPSS/PEEK after 24 h of incubation. This phenomenon was probably affected by considerable increase in the concentration of Ag^+^ ions resulting from the ICP-MS analysis (see Figure 5). Because the drop plate test is based on immersion of the sample in the medium with bacteria under statically-induced conditions, the concentration of Ag^+^ ions has a crucial impact on the display of the antibacterial effect. Our result is in agreement with the study of Ning et al. [39] who dealt with the determination of the MIC of Ag^+^ ions for *E. coli* at 2.5 10^−7^ mol/L, which is approximately 27 µg/L. Overall, these results indicate that the prepared samples manifested a strong antibacterial effect on *E. coli* after a long incubation period. Moreover, it follows from ICP-MS analysis that our surfaces may feed the environment with Ag ions at a sufficiently high level for a considerable period of time (at least one month) to maintain its antibacterial effect in medical-based applications.

## 3. Materials and Methods

### 3.1. Materials, Apparatus and Procedures

The preparation of AgNPs was accomplished electrochemically by immersion of two silver electrodes in sodium citrate according to [19]. The concentration of AgNPs in the resulting solution was measured by atomic absorption spectrometry (AAS), and for immobilization purposes, the Ag concentration in the AgNP colloid solution was set at 30 mg·L^−1^ by dilution with sodium citrate buffer (1 mM of sodium citrate in water). Subsequently, the size and shape of AgNPs was determined by transmission electron microscopy (TEM).

For the immobilization of AgNPs, poly(etherether ketone) foil (PEEK, thickness 50 µm, Goodfellow, Ltd., Huntington, UK) was chosen. This polymer is capable of changing the surface morphology when irradiated with laser light [40]. To transform the PEEK morphology into LIPSS, the polymer was irradiated with a KrF excimer laser (COMPexPro 50 F, Coherent, Inc., Santa Clara, CA, USA) according to the procedure described in our previous study [41]. Briefly, irradiation was performed through an aperture with an area of 5 × 10 mm^2^ with an incidence angle of 0° using a polarizing prism (model PBSO-248-100) under the following conditions: wavelength 248 nm, pulse duration 25 ns, 6000 pulses, laser fluence of 8 mJ·cm^−2^, repetition rate 10 Hz.

Immobilization of silver NPs was performed using the same instrumentation, KrF excimer laser (COMPex Pro 50F, Coherent, Inc., Silicon Valley, CA USA, wavelength 248 nm, pulse duration 25 ns, repetition rate 10 Hz, laser fluence of 10 mJ cm^−2^) on as-received PEEK and laser-pretreated PEEK with LIPSS structure. Polymer foil strip (30 × 8 mm) was cut and vertically placed in a central position of the spectroscopic cell (HellmaAnalitics GmbH, Mullheim, Germany, type No. 100-QS, light path 10 mm). Subsequently, 3.5 mL of colloidal AgNP solution was added using an automatic pipette. The laser light was linearly polarized with a UV-grade fused silica prism (model PBSO-248-100). The irradiation was performed perpendicularly to the polymer surface with an aperture of 5 × 10 mm^2^.

### 3.2. Analytical Methods

The silver concentration in the prepared colloid solution of AgNPs was determined by atomic absorption spectroscopy (AAS) on a Varian AA880 device (Varian Inc., Palo Alto, CA, USA) equipped with a flame atomizer at 242.8 nm wavelength. The typical error in this setup was less than 3%.

The visualization of silver NPs was performed using transmission electron microscopy (TEM) on a JEOL JEM-1010 device (JEOL Ltd., Akishima, Japan) operated at 400 kV. A colloidal sample was dropped on a copper grid coated with a thin amorphous carbon film placed on a filter paper. The solvent excess was removed and all samples were air-dried and kept under vacuum in a desiccator before the measurement. The particle size was determined from the TEM micrographs and calculated taking into account at least 500 particles.

The surface characteristics were measured by an atomic force microscope (AFM) on DimensionIcon (Bruker Corp., Billerica, MA, USA) in ScanAsyst-Air mode, providing surface morphology and roughness. A ScanAsyst mode using a silicon nitride tip (Bruker Corp., Billerica, MA, USA) was chosen, operating near its resonant frequency (70 kHz, spring constant 0.4 Nm^−1^). The scan speed was 0.5 Hz. The surface roughness (*R*_a_) represents the arithmetic average of the deviation from the center plane of a sample.

Additionally, the surface morphology of the samples was also visualized by a high-resolution FEGSEM microscope (MAIA3, TESCAN, Brno, Czech Republic), equipped with detectors of secondary (SE) and backscattered electrons (BSE). Samples were adhered with a double adhesive carbon tape (Cristine Groepl, Austria) to a brass stub and coated with a thin carbon layer in a vacuum evaporation device (JEE-4C; JEOL, Akishima, Japan). The samples were observed in UH Resolution mode at accelerating voltage of 3 kV, using an in-beam SE detector.

Atomic concentrations of silver Ag3d, carbon C1s, and oxygen O1s in silver-immobilized samples were determined by X-ray photoelectron spectroscopy (XPS). Measurement was performed using an Omicron Nanotechnology ESCAProbe P spectrometer (Omicron Nanotechnology GmbH, Taunusstein, Germany) at a pressure of 2 × 10^−8^ Pa. The X-rays were monochromated at 1486.7 eV with a step size of 0.05 eV. The collection of photoelectrons was carried out at a takeoff angle of 90, and 9° in the case of Ag/PEEK samples and 90, 9, and −9° in the case of Ag/LIPSS/PEEK samples to record any preferences in the decoration of PEEK in relation to the LIPSS structure. The scan size was 2 × 3 mm^2^. The CasaXPS software was used to evaluate measured spectra. The uncertainty of the measurement was less than 3%.

Inductively coupled plasma with a mass spectroscopy detector (ICP-MS) was used to determine the release rate of immobilized nanoparticles from the PEEK surface. Samples were placed in an Erlenmeyer flask, immersed in 50 mL of distilled water and left statically or on an orbital shaker for 28 days at room temperature. In specific time intervals (1, 3, 6, 12, 24 h, 7, 14, 21, 28 days), 2 mL of leachate was removed using an automatic pipette and analyzed. Since the primary objective of this analysis was to obtain information on the saturation concentration of Ag+ in the surrounding medium, the equivalent of the sample volume was always added in the form of distilled water, so the test volume was kept at 50 mL. The concentration of Ag was carried out on an Agilent 8800 triple-quadrupole spectrometer (Agilent Technologies, Santa Clara, CA, USA) equipped with an autosampler. Sample nebulization was performed using a MicroMist device equipped with a peristaltic pump. The measurements were performed in a high-energy mode, using a collision cell (He collision gas) to eliminate the effect of the adduct. The measurement uncertainty was less than 3%. The same device was also used to determine the total amount of biologically available silver (TABAAg). Samples with immobilized AgNPs, both planar and LIPSS, were immersed in 1 mL of concentrated nitric acid for 5 min. Subsequently, the polymers were removed, 19 mL of distilled water was added, and the samples were analyzed using ICP-MS.

The antibacterial effect of AgNP-immobilized PEEK was studied using a drop plate test based on the count of viable bacteria [42]. For testing, a Gram-negative *Escherichia coli* (DBM 3138) was used. For the preparation of inocula, bacteria were grown at 37 °C overnight, placed on an orbital shaker, and serially diluted in sterile physiological solution (PS). The optical density was measured at 600 nm (OD_600_). The PEEK, Ag/PEEK, and Ag/LIPSS/PEEK samples were immersed in 1 mL of PS inoculated with *E. coli* (1.1 × 10^4^ colony-forming units, CFUs). A PS comprising only *E. coli* was used as a control sample. The incubation was carried out under static conditions as follows: 24 °C, an incubation time of 3 and 24 h. The samples were prepared and analyzed in triplicate. Next, aliquots of 25 µL from each sample were vigorously vortexed and 5 drops were instilled on thirds of pre-dried agar plates (Luria-Bertani). After overnight cultivation on agar plates, the number of *E. coli* CFUs was counted and the mean values with the standard deviation were calculated. The whole experiment was carried out under sterile conditions.

## 4. Conclusions

This paper has shown that LIFT technology can be used successfully for the decoration of PEEK foil with AgNPs without affecting the specific surface morphology of the input polymer. These results broaden our understanding of incorporation of AgNPs into polymer surface, pretreated with a linearly polarized excimer laser, exhibiting periodic surface patterns known as LIPSS. We have shown that compared to the planar polymer, AgNP-decorated LIPSS significantly increases the concentration of silver in the surrounding environment within the first 24 h of mutual contact, although the concentration of immobilized particles on the surface of structured PEEK is approximately half that of the planar polymer. The antibacterial test against Gram-positive *E. coli* showed that the prepared samples manifested a strong antibacterial effect after 24 h of treatment, which is more pronounced in the case of a structured surface. We believe that nanostructured PEEK with embedded AgNPs may open up new possibilities in the production of templates for replication processes in the construction of functional bactericidal biopolymers or may be applied directly in tissue engineering because of the natural biocompatibility of PEEK and strong particle anchoring to the polymer surface.

## Figures and Tables

**Table 1:** Summary of surface characteristics derived from AFM analysis: surface roughness (*R*_a_), periodicity (*Λ*), and height (*h*).

Sample	*R*_a_ (nm)	*Λ* (nm)	*h* (nm)
PEEK	3.6	-	-
Ag/PEEK	4.2	-	-
Ag/PEEK_inset	6.7	-	-
LIPSS/PEEK	23.2	206.6	76.8
Ag/LIPSS/PEEK	25.7	205.3	81.1
Ag/LIPSS/PEEK_inset	26.4	205.6	81.3

## Data Availability

The data presented in this study are available on request from the corresponding author.
